# Recent advances in prognostication and treatment of polycythemia vera

**DOI:** 10.12703/r/10-29

**Published:** 2021-03-12

**Authors:** Bridget K Marcellino, Ronald Hoffman

**Affiliations:** 1 Division of Hematology and Medical Oncology, Tisch Cancer Institute, Icahn School of Medicine at Mount Sinai, One Gustave L. Levy Place, Box 1079, New York, USA

**Keywords:** Polycythemia vera, myeloproliferative neoplasm, treatment, prognostic tools

## Abstract

Polycythemia vera (PV) is a *BCR-ABL*–negative myeloproliferative neoplasm marked by acquisition of an activating mutation of *JAK2*, which leads to not only erythrocytosis but also frequently to leukocytosis and thrombocytosis, and is associated with a high symptom burden and increased thrombotic risk. PV has the potential to progress to myelofibrosis or an aggressive form of acute myeloid leukemia. Mutational profiling of patients with PV has led to the development of risk stratification tools to determine an individual’s risk of developing progressive disease. Although the current goals of PV treatment are to alleviate symptoms and reduce thrombotic risk, there are growing efforts to identify disease-modifying agents which will also prevent progression of disease. Here, we give an overview of the developing prognostic tools and therapeutic landscape for PV, focusing on four drug classes: pegylated interferon-alpha 2, MDM2 antagonists, hepcidin mimetics, and histone deacetylase inhibitors.

## Introduction

Polycythemia vera (PV) belongs to a group of chronic hematologic malignancies known as BCR-ABL–negative myeloproliferative neoplasms (MPNs). These diseases arise from a malignant hematopoietic clone gaining proliferative advantage over normal hematopoietic stem cells. Erythrocytosis is the defining feature in PV; however, owing to the driver lesion in the Janus-activated kinase 2 (*JAK2*) gene occurring at the hematopoietic stem cell level, multilineage involvement can result, leading to an overproduction of red cells, white cells, and platelets. Therefore, patients can also frequently exhibit leukocytosis and thrombocytosis in addition to erythrocytosis. The diagnosis of PV is made by meeting three major criteria of the World Health Organization (WHO): (1) hemoglobin of more than 16.5 g/dL in men or more than 16 g/dL in women or hematocrit of more than 49% in men or more than 48% in women or increased red blood cell mass, (2) bone marrow trilineage proliferation with pleomorphic mature megakaryocytes, and (3) presence of a *JAK2* mutation (*JAK2*V617F or a mutation at exon 12 of *JAK2*). It also can be diagnosed by meeting the first two major criteria and having a subnormal erythropoietin level^1^.

However, several investigators have suggested that the WHO criteria have important limitations. Red blood cell mass measurements are infrequently available throughout the world, making this parameter useful only at highly specialized centers. Alvarez-Larrán *et al*.^2^ and Ancochea *et al*.^3^ have presented data which indicate that hematocrit levels more consistently identify patients with a raised red blood cell mass than hemoglobin levels do. Hematocrit levels can be of limited value in diagnosing PV in pregnant females or patients presenting with splanchnic vein thromboses (SVTs). In addition, others view the routine use of marrow histopathology in establishing a diagnosis of PV to be of limited use^4^. In the British Society for Haematology Guideline for the diagnosis of PV (published in 2019)^4^, a pre-eminent group of British hematologists emphasized that several studies indicated that routine marrow sampling was frequently associated with a lack of consensus between pathologists examining the same specimens and that the use histopathology frequently was not suitable to establish a diagnosis of PV. They concluded that marrow histopathology was not mandatory in routine cases of PV but should be reserved for those patients with atypical clinical features, such as marked splenomegaly or a history of SVT, where it is necessary to establish whether there is an occult PV or to document evidence of progression of PV to myelofibrosis. The conclusions made by British hematologists reflect the standard of care at most institutions, whereas the WHO criteria have proven useful when identifying patients with PV for entry into clinical trials.

Patients with PV can either be asymptomatic or have myriad symptoms, including (most commonly) fatigue, pruritus, and night sweats^5^. Splenomegaly from extramedullary hematopoiesis is present at the time of diagnosis in about a third of patients but is only occasionally significant enough to result in abdominal pain and early satiety^6^. Arterial and venous thromboses are the main causes of morbidity and mortality in this patient population^7^. Additionally, about 20% of patients with PV progress to myelofibrosis or an aggressive form of acute myeloid leukemia (AML), known as MPN blast phase (MPN-BP)^8–10^.

The aims of the current treatment paradigm for PV are to reduce the risk of thrombosis and to alleviate symptoms by keeping the hematocrit to less than 45%^11^. First-line therapy for low-risk patients, who are defined as lacking a history of a thrombosis, is aspirin and phlebotomy. High-risk patients require aspirin and cytoreduction, most commonly with hydroxyurea or interferon (IFN). Recently approved second-line therapy is the JAK1/2 inhibitor ruxolitinib^12,13^. When compared with standard therapy in patients with PV resistant or intolerant to hydroxyurea, ruxolitinib was found to be a safe and effective drug to control hematocrit levels, reduce spleen volumes, and control symptoms. Current research is focused on identifying additional agents to reduce the risk of thrombosis and target the malignant stem cell. Here, we describe several new or advancing developments in improving the risk stratification systems and treatment options for PV.

## Mutational profiling in polycythemia vera

*JAK2*V617F is the driver mutation in more than 95% of PV cases, and nearly all other cases have a mutation in exon 12 of the *JAK2* gene^14,15^. In those patients with a clinical phenotype that resembles PV but lacks a *JAK2* driver mutation, a mutation in *LNK*, also called *SH2B3*, can be present. LNK plays an important role in hematopoiesis by negatively regulating JAK2 activation through its SH2 domain, thus inhibiting erythropoietin-receptor and MPL signaling^16^. LNK exon 2 mutations have been reported in patients with pure erythrocytosis^17^.

The role of additional myeloid mutations in the pathogenesis of PV is currently an area of great interest. Evaluation of whole blood or bone marrow DNA from PV patients with next-generation sequencing by using a 27-gene myeloid neoplasm panel revealed that about half harbored additional sequence variants or mutations^18^. Mutations can occur in several classes of genes: epigenetic modifiers (*DNMT3A*, *TET2*, and *ASXL1*), splicing factors (*SF3B1*, *SRSF2*, and *U2AF1*), metabolic enzymes (*IDH1* and *IDH2*), and tumor suppressors (*TP53*). *ASXL1* and *TET2* mutations are the most commonly found mutations in patients with PV after *JAK2*V617F. In a retrospective study of 100 patients with chronic MPN and either PV or essential thrombocythemia, mutations in *IDH1/2* or *SF3B1* were associated with myelofibrotic transformation and mutations in *ASXL1*, *TP53*, *SRSF2*, *IDH1/2*, and *RUNX1* were associated with transformation to MPN-BP^19^. Another analysis of 404 patients with PV revealed that *ASXL1*, *SRSF2*, and *IDH2* mutations were associated with poor outcomes due to lower overall, leukemia-free, and myelofibrosis-free survival^20^. A prognostic scoring system accounting for the presence of adverse spliceosome mutations has been established, but owing to the absence of PV course-modifying therapies, there has been no clear-cut evidence for the clinical utility of such prognostication^20^.

## Novel therapeutic avenues in polycythemia vera

### Pegylated interferon

IFN-α is a cytokine that regulates a multitude of biologic activities essential in cellular proliferation and differentiation^21^. The mechanisms that drive the activity of IFN-α in MPNs are not entirely understood but are attributed to its combined anti-inflammatory, immunomodulatory, anti-proliferative, and pro-apoptotic effects. Upon binding of IFN to IFN receptors, JAKs are activated and phosphorylate signal transducers and activators of transcription (STAT), which regulate the transcription of a broad array of genes^22^. This interaction with JAK-STAT signaling is of particular significance because of the constitutive activation of this pathway in patients with *JAK2*-mutated PV. There is evidence that the malignant clone driving PV can be eradicated with IFN treatment because of two proposed mechanisms: either its ability to exhaust the malignant stem cell pool by driving these cells out of quiescence to differentiate or its ability to activate the tumor suppressor TP53^23,24^.

Initial evidence that IFN-α2 suppresses myeloproliferation in PV was published in 1985^25^, and the role of IFN-α2 in the treatment of PV has been a topic of debate for over three decades. IFN can normalize blood counts and thereby reduce or eliminate phlebotomy requirements, alleviate symptoms, and decrease spleen size^26^. It is also hypothesized that IFN-α2 has the potential to induce molecular remissions in patients with PV^27^.

While small clinical studies have shown that treatment with IFN-α2 can produce clinical, hematologic, and molecular responses, the toxicities associated with IFN-α limited its clinical utility^28^. The most common adverse events of IFN are flu-like symptoms, local injection site reactions, headache, dizziness, activation of pre-existing autoimmune disorders, arthralgias, cytopenias, and liver and cardiac toxicity. IFN can also induce or exacerbate depressive and mania symptoms and therefore is contraindicated in patients with a history of psychiatric illness^29^. The advent of pegylated IFN compounds has allowed for less frequent dosing of IFN and therefore greater tolerability. Several clinical trials with such agents have shed light on the utility of pegylated IFN treatment in this patient population.

Studies performed by the MPN Research Consortium evaluated pegylated IFN-alfa-2a (Pegasys, Genentech, South San Francisco, CA, USA), which was administered subcutaneously weekly in three populations of patients with PV: (1) patients resistant or intolerant to hydroxyurea, (2) treatment-naïve patients, and (3) patients with a history of SVT. In a phase 2 trial of peginterferon in 50 PV patients with resistance or intolerance to hydroxyurea, the overall response rate at 12 months was 60%; 22% of patients had a complete remission and 38% had a partial remission^30^. A cohort of patients in this trial included 20 PV or essential thrombocythemia patients who had a history of SVT and who were required not to have had prior therapy with hydroxyurea. After 12 months of peginterferon therapy, the overall response rate was 70%, and none of the patients had a recurrence of SVT after a median follow-up of 2.2 years^31^. In a phase 3 randomized trial of peginterferon versus hydroxyurea in 168 high-risk patients with PV and essential thrombocythemia, peginterferon complete response rates were similar for the two treatment groups at 12 and 24 months, and peginterferon had a higher rate of grade 3/4 toxicity^32^.

Another phase 3 trial of another form of pegylated IFN, ropeginterferon-alfa-2b (PharmaEssentia, Burlington, MA, USA), revealed increased efficacy with longer duration of administration in patients with PV^33^. This form of pegylated IFN has favorable pharmacokinetic properties which allow it to be administered every two weeks. This study was a randomized, non-inferiority phase 3 trial of ropeginterferon-alfa-2 versus standard therapy in 306 patients with PV who either were hydroxyurea-naïve or had less than 3 years of hydroxyurea treatment. There was also an extension phase of the study. At 12 months, non-inferiority of ropeginterfron-alfa-2b to hydroxyurea when comparing hematological and spleen response was not apparent. At 36 months, however, the cohort that received ropeginterferon-2b had a significantly higher percentage of complete hematologic responses with decreased disease burden (53%) in comparison with the cohort that received hydroxyurea (38%).

Molecular responses evidenced by a reduction of *JAK2*V617F allele burden occurred with both ropeginterferon-alfa-2b and standard therapy in the first year of treatment. However, the allele burden progressively decreased after years 2 and 3 of ropeginterferon-alfa-2b treatment, whereas in the group that received hydroxyurea, the allele burden was initially reduced but with longer treatment the allele burden increased. This indicates that continued ropeginteferon-alfa-2b treatment has a sustained response on the malignant clone and has the potential to allow for prolonged remissions. As evidenced in these trials, the side-effect profiles of IFN versus hydroxyurea are disparate and are important when deciding between these agents. For instance, hydroxyurea is associated with an increased risk of dermatological malignancies whereas IFN can cause and worsen underlying psychiatric illness.

A phase II randomized clinical trial comparing ropeginterferon-alfa-2b versus phlebotomy alone in low-risk patients with PV is under way (ClinicalTrials.gov Identifier: NCT030030025). Interim analysis of patients in this study was recently reported^34^. Eighty-four percent of patients who received ropeginterferon-alfa-2b versus 60% in the phlebotomy group met the primary composite endpoint of percentage of patients maintaining a hematocrit of not more than 45% over 1 year without evidence of disease progression (*P* = 0.008), and the greatest evidence of response to ropeginterferon-alfa-2b appeared to occur after 6 months of treatment. Disease progression was evident in 8% of patients whose treatment was phlebotomy only, and there was no incidence of disease progression in the ropeginterferon-alfa-2b group. Additionally, ropeginterferon-alfa-2b was associated with an improvement in PV symptoms. It was also reported that a significant difference in ≥3 adverse events of grade ≥3 was not observed. Ropeginterferon-alfa-2b, therefore, appears to be safe and efficacious in low- or high-risk patients with PV. Whether peginterferon-alfa-2b therapy is indicated as frontline therapy in low-risk patients with PV will require the execution and completion of carefully controlled clinical trials with sufficient numbers of patients and long-term follow-up in order to determine its effects on long-term patient outcomes.

Long-term follow-up of the patients in these trials is vital to determine whether sustained remissions can be attained with this specific therapy. It remains uncertain whether IFN therapy in patients with PV reduces the incidence of thrombotic events or evolution to myelofibrosis or MPN-BP. However, in the aforementioned trials, peginterferon and ropeginterferon-alfa-2b have been shown to be non-inferior to hydroxyurea in terms of preventing thromboses. Whether the modest reductions in the variant allele burden achieved with either form of pegylated IFN are effective biomarkers for subsequent clinical events and disease progression has yet to be documented. One advantage of IFN therapy is the ability for the patients to enjoy drug holidays after they have achieved prolonged periods of hematological remissions. Unfortunately, in the overwhelming majority of such instances, evidence of the malignant clone reappears during these drug holidays.

There is increasing interest in combining pegylated IFN with ruxolitinib and other potentially active agents. A phase II study evaluating the combination of pegylated IFN-alfa-2a and ruxolitinib in PV and myelofibrosis was recently reported^35^. Of the 32 patients with PV treated with this combination, 31% attained remission and 9% achieved complete remission. Ten of the patients with PV dropped out of the study because of side effects likely from pegylated IFN-alfa-2a, again highlighting that a significant proportion of patients are unable to tolerate this therapy.

### MDM2 inhibition

There is accumulating evidence that dysregulation of the TP53 pathway plays a key role in the pathogenesis and progression of MPNs. TP53 is a tumor suppressor protein that responds to DNA damage by promoting DNA repair or inducing cell death^36^. Deletions and mutations of the *TP53* gene are present in a wide array of cancers, including MPNs, and loss or inactivation of this gene is associated with a poor prognosis^37^. Low-allele-burden *TP53* mutations are present without clear clinical consequence in a proportion of patients with PV^38^. *TP53* loss of heterozygosity, however, is associated with transformation of chronic leukemia to AML and about one fourth of MPN-related AML cases harbor a *TP53* mutation^39–41^.

Mouse double-minute homolog 2 (MDM2) and mouse double-minute homolog 4 (MDM4) are proteins that work in concert to negatively regulate the TP53 pathway^42^. They down-regulate TP53 activity via several mechanisms, including inhibition of *TP53* transcription and transactivation, promoting TP53 export from the nucleus and stimulating TP53 degradation. MDM2 is overexpressed in PV stem/progenitor cells, leading to decreased TP53 pathway activity^43^. Preclinical evidence indicated that MDM2 inhibition targets the *JAK2*V617F hematopoietic progenitor cells and this activity can be enhanced by combination therapy with low doses of pegylated IFN-α2a^44^.

Clinical evaluation of MDM2 inhibition in PV by using the class of drugs known as nutlins is ongoing. A phase 1 trial of the oral MDM2 antagonist in high-risk patients with treatment-refractory PV was performed by our group^45^. Patients initially received idasanutlin as a single agent; however, if a partial response was not reached after six cycles of treatment, combination therapy with low-dose pegylated IFN-α2a was allowed. Dose-limiting toxicity was not observed; however, gastrointestinal toxicity was evident in the majority of patients and frequently led to discontinuation of the drug. In terms of clinical activity, the overall response rates were 58% for idasanutlin monotherapy and 50% for combination therapy with idasanutlin and pegylated IFN-α2a. The *JAK2*V617F allele burden was reduced in all idasanutlin-treated patients except one, who exemplified resistance to idasanutlin and was found to have a baseline *TP53* mutation. On-target effects of idasanutlin were evidenced by elevated levels of plasma MIC-1, which is a serum biomarker for TP53 activation.

A phase 2 trial of the nutlin, KRT-232, in phlebotomy-dependent patients with PV is under way and evaluation of up to 320 patients with PV is planned (ClinicalTrials.gov Identifier: NCT0366996). For the dose escalation phase (2a), enrolled patients must have exhibited hydroxyurea resistance or intolerance or had prior treatment with IFN. In phase 2b of the trial, patients must be hydroxyurea-resistant or -intolerant and are randomly assigned to either KRT-232 or ruxolitinib. Primary endpoints are spleen reduction (at least 35%) and freedom from phlebotomy. Secondary endpoints are response rate, duration of response, and symptom improvement.

### Hepcidin mimetics

The majority of patients with PV are iron-deficient at diagnosis and therapeutic phlebotomy reduces iron levels further^46,47^. Although the benefits of phlebotomy are attributed mainly to the reduction in red blood cells, reducing levels of circulating iron is potentially contributory. Restriction of iron decreases hemoglobin synthesis and thereby could dampen malignant erythropoiesis; however, malignant stem cells may develop mechanisms to decrease sensitivity to iron deficiency, allowing erythroblasts to have a survival advantage in an iron-deficient state^48^. Iron deficiency can lead to non-hematological symptoms, including weakness, fatigue, cheilosis, pica, muscle dysfunction, and cognitive impairment. Many of the symptoms of iron deficiency overlap with symptoms previously attributed to PV, making it difficult to elicit their true etiology^49^. Repeated phlebotomies have the potential to worsen iron deficiency symptoms and have been shown to exacerbate disease-related symptoms such as pruritis^50^.

Hepcidin is a peptide hormone released by hepatocytes and mediates iron homeostasis primarily by inhibition of iron efflux through the iron transporter, ferroportin, which is found in erythrocytes, hepatocytes, and enterocytes^51^. It reduces plasma hemoglobin by decreasing intestinal absorption of dietary iron, promoting sequestration of iron by macrophages, and inhibiting release of iron stored in hepatocytes. Increased erythropoietic activity stimulates hepcidin suppression which normally results in increased absorption of intestinal iron and mobilization of iron from hepatic stores which allows for iron homeostasis. In PV, there are two competing factors in hepcidin regulation: expanded erythropoiesis, which decreases hepcidin levels, and inflammation, which increases hepcidin. Overall, patients with PV have suppressed hepcidin; however, for unclear reasons, this is insufficient to correct iron deficiency^48^.

Hepcidin agonists are under exploration in a variety of blood disorders, including PV^52^. These agonists come in a variety of forms, including full-length exogenous hepcidin, truncated forms of hepcidin (mini-hepcidins), endogenous hepcidin stimulators, ferroportin inhibitors, and ERFE antagonists. The goal of all these agents is to restrict systemic iron metabolism. Preclinical studies showed that treatment of *JAK2*V617F mouse models of PV with exogenous hepcidin led to normalized hematocrit levels and reduced splenomegaly with increased sequestration of iron in splenic macrophages^53^. It is hypothesized that in patients with PV requiring therapeutic phlebotomy, hepcidin suppression promotes iron absorption that enhances malignant erythropoiesis. However, treating with a hepcidin mimetic therefore should serve as a “chemical phlebotomy” by hindering excessive erythropoiesis, preventing systemic iron deficiency.

The hepcidin mimetic PTG-300 is being evaluated in patients with PV requiring phlebotomy in a phase 2 clinical trial (ClinicalTrials.gov Identifier: NCT04057040). Preliminary findings to date are promising, and PTG-300 is safe and tolerable. All 13 of the patients in the trial have maintained a hematocrit of less than 45% without phlebotomy for up to 7 months. Iron deficiency in these patients is being reversed as evidenced by increasing ferritin, iron, and transferrin saturation levels as well as normalization of red blood cell parameter, including mean corpuscular volume and mean corpuscular hemoglobin^54^.

### Givinostat

The expression of many genes involved in leukemogenesis is regulated by acetylation and deacetylation of histones. Preclinical studies showed that histone deacetylase inhibition could selectively target *JAK2*V617F mutant cells and down-regulate the JAK/STAT pathway in the malignant cell population^55^. Givinostat (Italfarmaco, Milan, Italy), a class I and II histone deacetyalse inhibitor, was evaluated in hydroxyurea-naïve and PV patients who received hydroxyurea and was found to be safe and tolerable^56,57^. A phase Ib/II trial of givinostat in PV confirmed safety and tolerability, established the maximum tolerated dose of givinostat to be 100 mg twice daily, and reported a high overall response rate^58^. The most common adverse events identified were diarrhea, thrombocytopenia, and increased blood creatinine. In part B of the trial, where the primary objective was to determine overall response after 3 months, 35 patients were enrolled and the overall response rate was 80.6%. The majority of patients had a hematological response and about a third of patients had symptom improvement, and pruritus was particularly targeted by this therapy. An international randomized clinical trial of givinostat versus hydroxyurea in high-risk patients with PV is planned.

## Conclusions

Ultimately, the goals of therapy in this chronic disease are to improve quality of life, reduce risk of thrombosis, and alter disease course. The therapeutic landscape of PV is evolving because of a more thorough understanding of the molecular mechanisms driving the pathogenesis and progression of this disease. Several classes of drugs being explored for these purposes include pegylated IFNs, MDM2 inhibitors, hepcidin mimetics, and histone deacetylase inhibitors. There is nascent evidence that these agents are fundamentally altering the disease, and clinical activity is evident particularly in inducing hematological and symptom responses. Further study is needed to determine whether these agents have a role in reducing thrombosis risk and preventing disease progression to myelofibrosis and MPN-BP. Given that PV is a chronic disease, it is essential that these therapeutics be safe and tolerable to allow for prolonged treatment. The future of PV treatment likely will require combination treatments, which are being explored in preclinical studies.
